# Infection prevention and control practices among primary healthcare nurses regarding COVID-19 in Saudi Arabia: A cross-sectional study

**DOI:** 10.1016/j.amsu.2022.103298

**Published:** 2022-02-22

**Authors:** Zammar Ayat, Al-Hamidi Sami

**Affiliations:** a General Directorate of Health Affairs, Supply Department, Al-Rabwa District, Bin Badis Street, Riyadh, Saudi Arabia; b King Saud University, Nursing College, Building 24, Second Floor, Riyadh, Saudi Arabia

**Keywords:** Infection prevention, Infection control, Practices, Primary healthcare nurses, COVID-19, Pandemic

## Abstract

**Background:**

Healthcare providers, particularly nurses, are at risk of infection as part of the COVID-19 pandemic since they assist in the disease's containment. By recognising the risk factors for infection and implementing suitable measures to reduce these risks, all reasonable efforts should be taken to control the spread of infection to them. The aim of the present study was to determine the level of infection prevention and control practises used by primary healthcare nurses in Saudi Arabia during the COVID-19 pandemic.

**Materials and methods:**

Cross-sectional design to examine the preventive practices of COVID-19 among healthcare professionals and community health nurses (n = 198) who worked in primary healthcare facilities in southwest Riyadh. Staff were randomly recruited, and a self-administered questionnaire was employed to collect demographic data, sources of COVID-19 information, and COVID-19 infection prevention and control measures.

**Results:**

Most of participants were male (57.6%), aged 30_39 years (50.5%), married (61.6), with monthly income of more than10,000 SAR, and more than half (74.0%) of the participants hold a diploma degree. Social media was the most prevalent source of information about COVID-19 (69.25%). About 91.4% used facemask in crowds and 65.2% of the participants wore medical Personal Protective Equipment during prescribing drugs. Furthermore, 94.5% of the participants wore a simple facemask and 32.8% washed their hands with running water and handwashing liquid. Females were most users of gloves and simple mask; who had a monthly income of 5000 SAR or more were the most who wore goggle mask and apron; non-nurse staff, aged 40_49 years and who had more than 10 years of experience were the most users of N95 respirator; while nurses, aged 30_39 years and with 6_10 years of experience were the most who used gowns. Participants' age, income, and work experience were all found to be important factors linked with COVID-19 infection prevention and control practices.

**Conclusion:**

Overall practicing preventive measures by Healthcare professionals in Saudi primary healthcare centres to encounter COVID-19 pandemic were generally significant. Policymakers at the Ministry of Health should monitor preventive practises regarding COVID-19 infection among all healthcare providers in other facilities. It is recommended to conduct systematic reviews and amend current guidelines for preventive practices in the COVID-19 pandemic to promote maximum state among population.

## Highlights

• What is already known on this topic.
  • In the wake of MERS-CoV and SARS-CoV epidemics, experts have created measures and criteria for the prevention and treatment of COVID-19 epidemic.
  • COVID-19 infection prevention and control practises have not been studied in basic healthcare settings in Saudi Arabia in the present time.
• What this study adds.
  • Health care workers at Saudi primary healthcare centres were typically taking preventative steps to deal with the COVID-19 pandemic, which was noteworthy.
  • COVID-19 infection prevention practises at other healthcare institutions should be taken into account by the Ministry of Health's policymakers.

## 1 Introduction

Viral illnesses continue to emerge over the world, and these diseases are regarded to be important public health hazards. Epidemics such as severe acute respiratory syndrome coronavirus (SARS-CoV), Middle East respiratory syndrome coronavirus (MERS-CoV) and H1N1 influenza were emerged in the last 12 years [1]. Since the beginning of this year, there have been 313 million confirmed cases of new coronavirus illness (COVID-19) worldwide, with a rising mortality toll of 5.5 million cases [2]. Saudi Arabia has had 588,183 confirmed cases of COVID-19, with 8897 deaths as a result of the disease [3].

Due to a loss in awareness of the dangers of communicable illnesses and a lack of compliance with infection prevention and control methods, nurses and other healthcare professionals face an increasing risk of exposure to new and re-emerging infectious diseases [4]. As the first line of defence in the fight against an outbreak, healthcare workers, such as nurses, are particularly vulnerable to infection. Consequently, all reasonable precautions must be done to prevent the transmission of the virus to staff, first by identifying the risk factors for infection, and then by adopting suitable measures to limit these risks [5].

Overcrowding, a lack of isolation rooms, and pollution of the environment are all known risk factors for the disease's spread among healthcare workers. As a result, some nurses may have a poor understanding of infection control procedures [6]. An infectious illness can impact nurses' attitudes and actions in ways that directly increase patient risk of infection [7]. Understanding nurses' knowledge, attitudes, and behaviours on infection and preventive methods and practises might assist anticipate the effects of planned behaviour among them. The major objective is to find out how well Saudi Arabian primary healthcare nurses were able to prevent and manage infection during the COVID-19 pandemic.

Recent research in under-developed countries have found a low level of compliance with appropriate infection control procedures and basic precautions, while in developed countries, a lack of proper control methods, resources, and regulation has been noted as a large and significant issue that created a race towards infection management [8]. Measures and preventive standards for COVID-19 infection and prevention have been developed based on experience and knowledge gained while responding to outbreaks such as MERS-CoV or SARS-CoV [9].

According to existing data, SARS-CoV-2 is genetically identical to SARS-, but it has a distinct mode of transmission and other features [10,11]. As a result, there is a critical need to improve nurses' knowledge and behaviours addressing COVID-19-specific infection prevention and control methods. This information must be accessible in order to take a significant step towards evaluation.

Furthermore, current studies have not assessed nurses' knowledge and practises regarding infection prevention and control practises for COVID-19 in primary healthcare facilities in Saudi Arabia, which could result in an increase in disease transmission due to non-adherence to these measures and practises. Specific objectives were:

1. To identify the sources of knowledge and associated information among nurses on infection control strategies for COVID-19.
2. To examine community healthcare nurses' infection prevention and control methods during the COVID-19 pandemic.
3. To evaluate the relationship between the infection prevention practices of primary healthcare nurses and their demographic characteristics.

## 2 Methods

This cross-sectional study has been reported in line with the STROCSS criteria [12], and was carried out with the registration code NCT05130749, which is available at http://clinicaltrials.gov/, under the direct supervision and direction of the US National Institute of Health (NIH). Study approval was received from King Saud University's ethics committee approval (No: KSU-HE-21-35, Date: 02-02-2021).

The target population included healthcare practitioners and community health nurses who worked at the aforementioned centres, as there were 800 nurses in total. The determined sample size was 207 healthcare providers, and the researcher recruited study participants from primary healthcare centres using a straightforward sampling strategy. The current study included male and female healthcare practitioners with at least one year of experience and who worked in all areas of primary healthcare facilities during the COVID-19 pandemic. Healthcare providers that are unwilling to participate in the present study have been eliminated. In this study, 198 out of 207 participants (95.6%) answered the survey questionnaire. This response rate is regarded to be outstanding, as the researcher actively urged study respondents to participate.

This study used a quantitative cross-sectional design, and was conducted at primary health care clinics located in southwest Riyadh (*n = 25* governmental clinics). Sample of the study was calculated using Stephen Thompson formula (α = 0.05, C.I. 95.0%, N = 450). The following formula was used to calculate the sample:

n= (450x 0.50(1–0.50))/ (450-1x (0.0025/3.84) +0.50(1–0.50)) = 207

The calculated sample size is 207 healthcare providers. The researcher used a convenient sampling method to recruit study participants from the primary healthcare centres. In this study, 198 out of 207 (95.6%) have responded to participate in the study questionnaire. This response rate is considered excellent since the researcher encouraged the study participants to participate in the study.

### 2.1 Research instrument

Data were collected from community health nurses using a questionnaire that was adapted from other researchers [[13], [14], [15]]. The questionnaire's first section asked for demographic data such as gender, age, marital status, socio-economic information, the name of the primary healthcare centre, and level of education. The second section comprised sources of knowledge on COVID-19; respondents replied on a scale of 1 least used (1), occasionally (2), more frequently (3), and most used (4). The third section had six items pertaining to nurses' infection prevention and control methods with reference to COVID-19, and respondents replied on a scale of yes (2), occasionally (1), and no (0). Permission to use the questionnaire has been requested from the author and is awaited.

In order to get feedback on the questions and make sure the questionnaire was reliable, a pilot study with 30 healthcare practitioners was done. For Face and content validity, the questionnaire was reviewed and confirmed to be adequate by a panel of professional nurses with expertise and understanding of research methodologies. For Instrument reliability, Cronbach's coefficient alpha was used to determine reliability coefficients for the majority of uses.

### 2.2 Data analysis

Demographic data, such as age, gender, experience, and educational level, were maintained as categories and numerical variables. The demographics were coded numerically in the questionnaire. To address the study topics and analyse the connection between demographic data and nurses' knowledge, attitude, and practise, inferential statistics such as the independent sample *t*-test and one-way ANOVA were utilised. Significance level was set at p < 0.05 value indicating a significant connection.

## 3 Results

### 3.1 Participants’ demographic

Of all 198 respondents, 114 (57.6%) were male, while 84 (42.4%) were female respectively. Healthcare workers were more likely to be married (61.6%) than being single (34.9%) or divorced (2.5%). A total of 100 people (50.5%) and 162 people (81.8%) were in the 30_39 age range and had a monthly salary of at least 10,000 Saudi riyals, respectively. Non-nurses comprised 68.7% of the workforce, while those with 0_5 years of experience comprised 42.9%. According to the data, more than half (74.0%) of the participants in this survey held a diploma (less than a bachelor's degree), whereas 26.0% held a bachelor's degree or higher. Total of 136 (68.7%) of staff were non-nurses, while 85 (42.9%) had 0_5 years of experience. Demographic data of the study participants were shown in Table 1.

**Table 1:** Sample distribution according to the participants’ demographics.

**Variables**	Number	Percentage (%)
**Gender**		
Male	114	57.6
Female	84	42.4
**Age Groups**		
<30 years	80	40.4
30–39 years	100	50.5
40–49 years	14	7.1
50 years or more	4	2.0
**Marital Status**		
Married	122	61.6
Single	71	35.9
Divorced	5	2.5
Educational level		
< Bachelor degree	146	74.0
≥ Bachelor degree	52	26.0
**Income**		
<5000 SAR	11	5.6
5000 - <10000	25	12.6
≥10000	162	81.8
**Job Category**		
Nurses	62	31.3
Others	136	68.7
**Years of Experience**		
0–5 years	85	42.9
6–10	36	18.2
>10 years	77	38.9

Social media was the most often cited source (69.25%), followed by seniors and other co-workers (61.25%). Newspapers and magazines were the least often cited sources (44.75%), as shown in Table 2.

**Table 2:** Mean, SD and percentage of participants’ source of information regarding COVID-19.

Source	Mean	SD	%
**Social Media**	2.77	0.98	69.25
**Seniors & Other Colleagues**	2.45	0.95	61.25
**Seminars & workshops**	2.30	0.99	57.50
**Radio & television**	2.15	0.99	53.75
**Posters & Pamphlets**	2.11	0.94	52.75
**Newspapers & Magazines**	1.79	1.00	44.75

Analysis of preventive practices measures in COVID-19 pandemic indicated that wearing a facemask in crowds (91.4%) was more prevalent than other practices; such as discarding used tissues (87.4%), covering noses and mouths with tissues while sneezing or coughing (85.9%), or even washing hands constantly with soap or hand sanitizer (83.8%). According to data in Table 3, avoiding touching eyes, nose or mouth as far as possible (76.3%) was more frequent than of staff educating patients about the disease (69.7%).

**Table 3:** Frequency, mean, and percentage of participants’ preventive practices measures in COVID-19 pandemic.

Participants' Practices	No (%)	Sometimes (%)	Yes (%)
**Do you educate your patient about the disease?**	11 (5.6)	49 (24.7)	**138(69.7)**
**Do you use facemask in crowds?**	3 (1.5)	14 (7.1)	**181 (91.4)**
**Do you avoid touching your eyes, nose or mouth as far as you can?**	9 (4.5)	38 (19.2)	**151 (76.3)**
**Do you throw the used tissue in the trash?**	4 (2.0)	21 (10.6)	**173 (87.4)**
**Do you cover your nose and mouth with a tissue during sneezing or coughing?**	0 (0.0)	28 (14.1)	**170 (85.9)**
**Do you use soap or hand sanitizer to wash your hands continuously?**	2 (1.0)	30 (15.2)	**166 (83.8)**

**Fig. 1:**
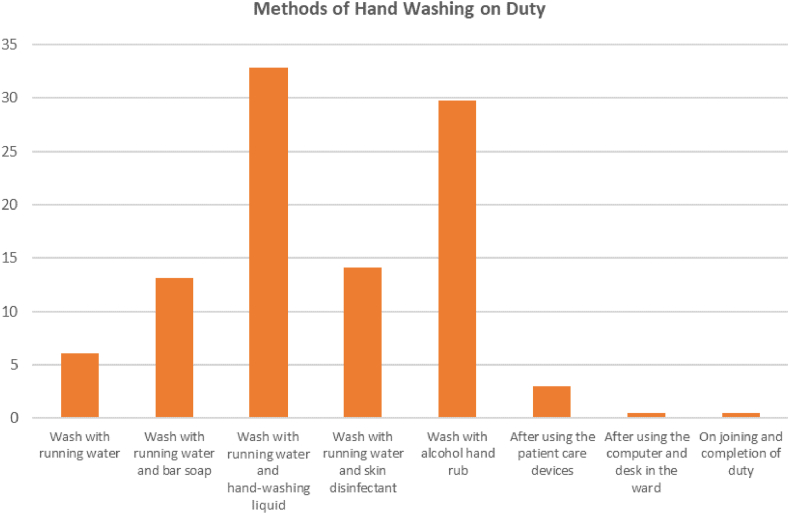
Methods which are Used by the Participants when Washing their Hands on Duty.

**Fig. 2:**
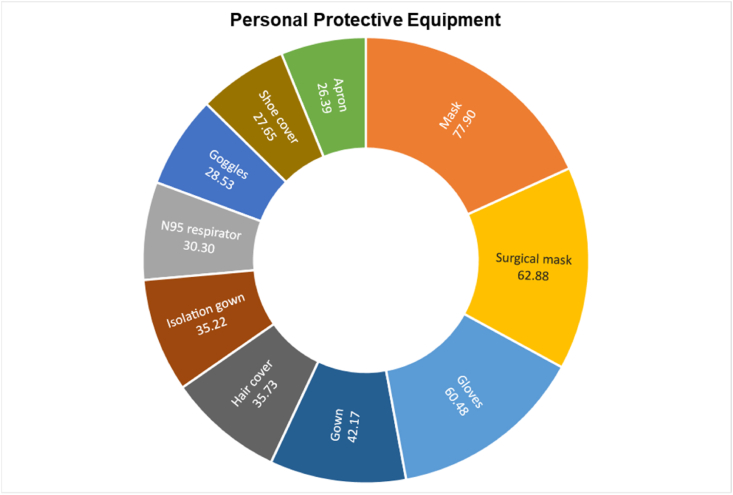
Personal protective equipment.

According to the analysis, using running water and handwashing liquid (32.8%) was the most reported method of hand washing practiced on duty, compared to using only with running water (6.1%), as visualized in Fig. 1. Also, most of participants (65.2%) wore medical PPE during prescribing drugs, compared to only 11.6% who wore medical PPE when making clinical rounds. Regarding other PPE practices, as illustrated in Fig. 2, washing hands before meals was done by the majority of staff (98.5%), while the least PPE practiced was wearing a gown (42.17%).

Of all PPE practices, One-Way ANOVA analysis showed a statistically significant mean scores of practises among staff aged 30–39 years (t/f (3, 194) = 3.952, p = 0.009). Respectively, earning more than 10,000 Saudi Riyal (t/f (3, 194) = 4.672, p = 0.01) and having more than 10 years in experience (t/f (3, 194) = 9.387, p < 0.001) had a substantially greater mean level of PPE practises. Demographic data of the study participants was shown in Table 4.

**Table 4:** Differences in the Participants Practices about Preventive Measures about COVID-19 with regard to their Demographics.

Preventive Measures	N	Mean	SD	t/f statistics (df)	p value^1^
Gender					
Male	114	1.77	0.30	−1.465 (196)	0.145^a^
Female	84	1.83	0.24		
Age groups					
<30 years	80	1.72	0.31	3.952 (3, 194)	0.009^b^
30–39 years	100	1.85	0.19		
40–49 years	14	1.83	0.39		
50 years or more	4	1.62	0.43		
Marital status					
Married	122	1.82	0.27	2.845 (2, 195)	0.059^b^
Single	71	1.74	0.28		
Divorced	5	1.96	0.07		
Income					
<5000 SAR	11	1.56	0.41	4.672 (2, 195)	0.010a
5000- <10000 SAR	25	1.78	0.30		
≥10000	162	1.81	0.25		
Work experience					
0–5 years	85	1.72	0.30	9.387 (2, 195)	0.000a
6–10 years	36	1.74	0.28		
> 10 years	77	1.90	0.20		
Job category					
Nurses	62	1.85	0.25	3.590 (2, 196)	0.060b
Others	136	1.77	0.28		

a Independent sample *t*-test.

b One-Way ANOVA.

Wearing gloves had a considerably higher mean score among female participants than among male participants (p = 0.003). Staff aged 30–39 years old had a considerably greater degree of wearing gloves than the others and those with an income of more than 10,000 SAR (p < 0.01). When it comes to wearing a simple mask, females mean score (p = 0.032) was substantially greater than male participants. Those with incomes of 10,000 SAR or more were the most who used simple masks (p < 0.001). Respectively, staff 30–39 years of age, with more than 10 years of experience and being a nurse (p < 0.001) were the most who used N95 respirators.

Data showed that those with an income of more than 100,000 SAR had a substantially greater mean level of surgical mask use than those with an income of less than 10000 SAR (p = 0.032). The mean score of participants' practises on using a surgical mask did not change significantly depending on their level of expertise or job type (p > 0.05). A comparison of using goggles and with those with an income of more than 10,000 SAR showed a significant difference in the mean score (p < 0.01).

Wearing an apron was significantly most prevalent among staff earning 5000_10,000 SAR (p = 0.004), while depending on gender, age, marital status, level of expertise or job type were found not significant (p > 0.05). A look at the results indicated that those 30–39 years old (p = 0.01), who earned more than 10000 SAR (p < 0.001) and those who have 10 years of experience had a greater mean level of gown use compared to other groups. To make matters more interesting, the average of gown use among nurses seemed to be much greater than that among other healthcare personnel Table 5.

**Table 5:** Differences in the Participants Practices about Personal Protective Equipment with regard to their Demographics.

Demographics	Gloves					Simple Mask					N95 Respirator					Surgical Mask				
	N	Mean	SD	t/f (df)	p^1^	N	Mean	SD	t/f (df)	P	N	Mean	SD	t/f (df)	p	N	Mean	SD	t/f (df)	p
Gender																				
Male	114	2.17	1.43	−2.989 (196)	0.003^a^	114	2.94	1.43	−2.163 (196)	0.032a	114	1.18	1.06	−0.414 (196)	0.679^a^	114	2.35	1.55	−1.688 (196)	0.093^a^
Female	84	2.75	1.19			84	3.34	1.03			84	1.25	1.14			84	2.72	1.44		
Age Groups																				
<30 years	80	2.20	1.30	5.497 (3, 194)	0.001^b^	80	3.07	1.29	2.547 (3, 194)	0.057^b^	80	0.81	0.81	7.785 (3, 194)	0.000^b^	80	2.28	1.51	2.488 (3, 194)	0.062^b^
30–39 years	100	2.75	1.27			100	3.28	1.15			100	1.50	1.16			100	2.79	1.42		
40–49 years	14	1.64	1.69			14	2.42	1.74			14	1.64	1.39			14	1.92	1.81		
50 years or more	4	1.25	1.25			4	2.25	2.06			4	0.50	0.57			4	2.25	2.06		
Marital Status																				
Married	122	2.35	1.36	1.034 (2, 195)	0.357^b^	122	3.09	1.36	0.067 (2, 195)	0.936^b^	122	1.25	1.11	0.372 (2, 195)	0.690^b^	122	2.48	1.57	0.286 (2, 195)	0.752^b^
Single	71	2.47	1.37			71	3.15	1.19			71	1.12	1.10			71	2.53	1.43		
Divorced	5	3.20	1.09			5	3.20	1.09			5	1.40	0.54			5	3.00	1.41		
Income																				
<5000 SAR	11	1.09	1.37	5.972 (2, 195)	0.003^a^	11	1.63	1.43	8.212 (2, 195)	0.000^a^	11	1.00	0.63	2.297 (2, 195)	0.103^a^	11	1.36	1.43	3.508 (2, 195)	0.032^a^
5000_10000 SAR	25	2.64	1.11			25	3.20	1.00			25	1.64	1.25			25	2.48	1.19		
≥10000	162	2.47	1.35			162	3.20	1.26			162	1.16	1.09			162	2.59	1.54		
Work Experience																				
0–5 years	85	2.41	1.29	0.243 (2, 195)	0.784^a^	85	3.08	1.23	1.798 (2, 195)	0.168^a^	85	0.88	0.89	7.091 (2, 195)	0.001^a^	85	2.41	1.50	0.462 (2, 195)	0.631^a^
6–10 years	36	2.55	1.27			36	3.47	0.99			36	1.44	1.10			36	2.69	1.41		
> 10 years	77	2.36	1.48			77	2.98	1.44			77	1.46	1.22			77	2.54	1.58		
Job Category																				
Nurses	62	2.67	1.26	1.810 (196)	0.072^b^	62	3.19	1.22	0.569 (196)	0.570^b^	62	1.66	1.18	4.020 (196)	0.000^b^	62	2.67	1.39	1.016 (196)	0.311^b^
Others	136	2.30	1.39			136	3.08	1.32			136	1.00	0.99			136	2.44	1.56		

Demographics	Goggles Mask					Apron					Gown				
	**N**	**Mean**	**SD**	**t/f (df)**	**p**^**1**^	**N**	**Mean**	**SD**	**t/f (df)**	**p**	**N**	**Mean**	**SD**	**t/f (df)**	**p**
Gender															
Male	114	1.06	1.09	−1.085 (196)	0.279^a^	114	1.05	1.08	−0.042 (196)	0.966^a^	114	1.60	1.31	−1.017 (196)	0.310^a^
Female	84	1.25	1.35			84	1.05	1.19			84	1.79	1.31		
Age Groups															
<30 years	80	0.88	1.04	2.425 (3, 194)	0.067^b^	80	0.88	1.04	1.595 (3, 194)	0.192^b^	80	1.37	1.24	3.889 (3, 194)	0.010^b^
30–39 years	100	1.31	1.27			100	1.17	1.128			100	1.97	1.30		
40–49 years	14	1.50	1.45			14	1.35	1.54			14	1.71	1.43		
50 years or more	4	0.75	0.95			4	0.50	1.00			4	0.75	0.95		
Marital Status															
Married	122	1.18	1.17	0.351 (2, 195)	0.704^b^	122	1.02	1.10	0.298 (2, 195)	0.742^b^	122	1.6557	1.27	0.298 (2, 195)	0.742^b^
Single	71	1.05	1.30			71	1.08	1.20			71	1.7183	1.40		
Divorced	5	1.40	0.54			5	1.40	0.54			5	2.0000	1.22		
Income															
<5000 SAR	11	0.90	1.04	5.656 (2, 195)	0.004^a^	11	1.27	0.90	5.633 (2, 195)	0.004^a^	11	1.09	1.04	9.633 (2, 195)	0.000^a^
5000_10000 SAR	25	1.88	1.36			25	1.72	1.24			25	2.68	1.10		
≥10000	162	1.04	1.16			162	0.93	1.09			162	1.57	1.29		
Work Experience															
0–5 years	85	0.92	1.14	2.659 (2, 195)	0.073^a^	85	0.91	1.11	1.132 (2, 195)	0.324^a^	85	1.37	1.28	6.439 (2, 195)	0.002^a^
6–10 years	36	1.16	1.10			36	1.19	1.14			36	2.27	1.27		
> 10 years	77	1.36	1.29			77	1.14	1.14			77	1.75	1.27		
Job Category															
Nurses	62	1.3226	1.23	1.427 (196)	0.155^b^	62	1.27	1.14	1.846 (196)	0.066^b^	62	2.06	1.19	2.775 (196)	0.006^b^
Others	136	1.0588	1.19			136	0.95	1.11			136	1.51	1.33		

1 significant, p < 0.05,a One-Way ANOVA, b Independent sample *t*-test.

## 4 Discussion

This is a cross-sectional study on 198 primary healthcare staff working in southern Riyadh, Saudi Arabia that aimed to identify their sources of knowledge regarding infection control strategies for COVID-19, examine their infection prevention and control methods during the pandemic and to reflect the relationship between the infection prevention practices of primary healthcare nurses and their demographic characteristics.

This study indicated that social media was the most prevalent source of knowledge on COVID-19, in accordance to Saqlaina et al. [13] who found that the media is the primary source of knowledge on COVID-19 in the United States, and also consistent with the findings of Wang et al. [16], who found that COVID-19 was widely disseminated over the internet and social media. Many individuals, including nurses and other healthcare practitioners, regard media sources like Facebook and WhatsApp to be the primary sources for information. This might be a contributing factor in the current study's findings. Other types of social media, such as Facebook and Twitter, were deemed more convenient than reading newspapers and magazines when it comes to be educated about pandemic diseases.

Face masks have been shown to be the most commonly used technique of preventing infection among healthcare workers and nurses was using a facemask in a crowd (91.4%) than other practises, this new finding was in line with those findings in Deressa et al. [17] who found that the most common practise among healthcare workers was to use facemasks. Also, these findings were similar with the current findings. as previously reported by Zhang et al. [18] who found that 89.7% of health care professionals followed the right procedures for infection and preventive control in regard to COVID-19 pandemic. On the other hand, Saqlain et al. [13] reported that hand washing with soap was the most common good habit (96.1%). Furthermore, the current results differed from those of previous studies by Nour et al. and Khan et al. [19,20] that found that 95.4% and 85.7% of healthcare providers had washed their hands, respectively.

Based on the results of the present study, 82% of healthcare staff educated their patients about COVID_19 infection, this suggests that the staff at this hospital were just not providing enough health education to avoid COVID-19 infection on a daily basis. The findings of Saqlain et al. [13] suggested that 95.4% of participants educated their patients about the condition, which is not consistent with this study's findings.

According to current findings, 77.9% of participants used a simple mask; 62.8% of those used surgical masks; 60.4% wore gloves; and 42% wore gowns. These PPE were significantly more practiced than in Oladele et al. [21], as 20.6% of nurses used facemasks. Most recommendations for infection prevention and control encouraged the use of surgical masks and N95 respirators for all aerosol-transmitted operations by health care workers, hence the participants in this research were more likely to use simple facemasks on a regular basis [5].

In the current study, there was a substantial correlation between age and COVID-19 preventative measures, with the 31-39-year-old age group being a key determinant for excellent practise against COVID-19 pandemic, same as for Saqlain et al. [13] who showed that health care practitioners in this age group were characterised by a high level of activity and attention to preventative measures, so it might have a considerable impact on the average degree of practises for this age group, with no significant correlation between gender and behaviours related to COVID-19 preventative measures when looking at participants' gender and their preventive measures.

This study found that those earning between 5000 and 10,000 SAR had a significantly greater mean level of PPE practices than those earning below 5000. Those in this income bracket were more likely to stick around because they were more satisfied than those in lower-income groups.

Compared to healthcare professionals with less than 10 years of experience, healthcare providers with more than 10 years of experience were more knowledgeable and aware of COVID-19 prevention measures, leading to greater adherence to COVID-19 prevention procedures. Healthcare practitioners with fewer than 5 years of experience, on the other hand, require further training in preventative measures. Similarly, Saqlain et al. [13] found that experience and behaviours related to COVID-19 prevention were linked.

In this study, preventative measures practices regarding COVID-19's did not change significantly based on their job classification. However, this study's findings were not in line with those of Saqlain et al. [13], who found a correlation between job category and behaviours related to COVID-19 prevention. As a result, just 31.3% of participants were nurses, while 68.7% were other healthcare workers; this indicated that the two professions were not interchangeable.

## 5 Conclusion

Healthcare practitioners' overall procedures involving COVID-19 were acceptable, according to the findings of the research. Preventive measures, such as wearing PPE, should be a higher priority for primary care nurses and other healthcare personnel. Those who adhere to these preventative measures should serve as role models for patients and the general public. PPE, comprising gloves, apron, gown, and mask, should be utilised by healthcare personnel. In addition, nurses and other healthcare professionals should familiarise themselves with COVID-19 practises. They need to put this new information into practise on a regular basis.

To ensure primary healthcare workers' compliance with safety at work at this unprecedented moment, there are a number of recommendations based on this research. Providing suitable and quality-compliant personal protective equipment (PPE) and a safe working environment to frontline healthcare workers will assist reduce the psychological stress caused by this pandemic while also retaining the healthcare workforce.

### 5.1 Limitations

Nurses and other healthcare professionals had been the focus of this research. In this study, there were no distinct categories of healthcare professionals, which makes it impossible to compare the results to other groups. In addition, the number of nurses in this study is quite low when compared to that of other types of healthcare professionals, and this small number of nurses would not reflect the opinion of the overwhelming number of nurses in PHC in KSA. Although this study used a self-reported questionnaire to collect data, alternative approaches may have been more accurate in determining the amount of practise of participants.

### 5.2 Implications for health policy and primary healthcare

COVID-19's preventative measures should be monitored by health officials in primary care and the ministry of health, and the process of compliance should be monitored in-service. In order to raise the knowledge of healthcare practitioners regarding these behaviours, educational and training methods must be used. There should be a greater focus on healthcare practitioners with less than six years of experience. Those who earn less than 5000 SAR should also be considered. Nurses and other healthcare workers should be encouraged to use personal protection equipment by policymakers. Personal protection equipment for nurses and other healthcare workers should be provided to primary healthcare facilities by the ministry of health.

## Consent

Written informed consent was obtained from the patient for publication of this case report and accompanying images. A copy of the written consent is available for review by the Editor-in-Chief of this journal on request.

## Provenance and peer review

Not commissioned, externally peer-reviewed.

## Sources of funding

There are no sources of funding for this research project.

## Ethical Approval

Ethical Approval has been taken from King Saud University.

No: KSU-HE-21-35.

Date: 02-02-2021.

## Author contribution

Ayat Mohammad Zammar (AM): Conducted the literature search and wrote the paper.

Sami Abdul Rahman Al-Hamidi (SA): Editing of writing.

## Registration of research studies

1. Name of the registry: http://clinicaltrials.gov/
2. Unique Identifying number or registration ID: NCT05130749
3. Hyperlink to your specific registration (must be publicly accessible and will be checked): https://clinicaltrials.gov/ct2/show/NCT05130749?term=NCT05130749&draw=2&rank=1

## Guarantor

Ayat Mohammad Zammar Sami Abdul Rahman Al-Hamidi.

## Declaration of competing interest

There are no conflicts of interest between authors of this manuscript.
